# Clinical Characteristics and Diagnostic Challenges of COVID−19: An Update From the Global Perspective

**DOI:** 10.3389/fpubh.2020.567395

**Published:** 2021-01-11

**Authors:** S. M. Hasan Israfil, Md. Moklesur Rahman Sarker, Parisa Tamannur Rashid, Ali Azam Talukder, Khandkar Ali Kawsar, Farzana Khan, Selina Akhter, Chit Laa Poh, Isa Naina Mohamed, Long Chiau Ming

**Affiliations:** ^1^Department of Pharmacy, State University of Bangladesh, Dhaka, Bangladesh; ^2^Pharmacology and Toxicology Research Division, Health Med Science Research Limited, Dhaka, Bangladesh; ^3^Department of Microbiology, Jahangirnagar University, Savar, Bangladesh; ^4^Department of Neurosurgery, St. Georges Hospital, London, United Kingdom; ^5^Department of Public Health, Daffodil International University, Dhaka, Bangladesh; ^6^Centre for Virus and Vaccine Research, School of Science and Technology, Sunway University, Petaling Jaya, Malaysia; ^7^Department of Pharmacology, Faculty of Medicine, Universiti Kebangsaan Malaysia (The National University of Malaysia), Cheras, Malaysia; ^8^PAPRSB Institute of Health Sciences, Universiti Brunei Darussalam, Bandar Seri Begawan, Brunei

**Keywords:** clinical characteristics, SARS coronavirus (SARS-CoV-2), novel coronavirus diseases (COVID-19), diagnostic challenges, systematic review

## Abstract

Clinical characteristics are essential for the correct diagnosis of diseases. The current review aimed to summarize the global clinical characteristics of the COVID-19 patients systematically and identify their diagnostic challenges to help the medical practitioners properly diagnose and for better management of COVID-19 patients. We conducted a systematic search in PubMed, Web of Science, Scopus, Science Direct, and Google Scholar databases for original articles containing clinical information of COVID-19 published up to 7th May 2020. Two researchers independently searched the databases to extract eligible articles. A total of 34 studies from 8 different countries with 10889 case-patients were included for clinical characteristics. The most common clinical symptoms were cough 59.6, fever 46.9, fatigue 27.8, and dyspnea 20.23%. The prominent laboratory findings were lymphocytopenia 55.9, elevated levels of CRP 61.9, aspartate aminotransferase 53.3, LDH 40.8, ESR 72.99, serum ferritin 63, IL-6 52, and prothrombin time 35.47%, and decreased levels of platelets 17.26, eosinophils 59.0, hemoglobin 29, and albumin 38.4%. CT scan of the chest showed an abnormality in 93.50% cases with bilateral lungs 71.1%, ground-glass opacity 48%, lesion in lungs 78.3%, and enlargement of lymph node 50.7%. Common comorbidities were hypertension, diabetes, obesity, and cardiovascular diseases. The estimated median incubation period was 5.36 days, and the overall case fatality rate was 16.9% (Global case fatality outside China was 22.24%: USA 21.24%, Italy 25.61%, and others 0%; whereas the case fatality inside the Hubei Province of China was found to be 11.71%). Global features on the clinical characteristics of COVID-19 obtained from laboratory tests and CT scan results will provide useful information to the physicians to diagnose the disease and for better management of the patients as well as to address the diagnostic challenges to control the infection.

## Introduction

In late December 2019, the Chinese government officially announced the novel coronavirus (2019-nCoV) outbreak in Wuhan. To contain the virus, Wuhan and eventually the whole Hubei province was put into massive lockdown. The virus, which was later named SARS-CoV-2, got out anyway in other countries of the world. Although limited to China initially, the novel strain of the coronavirus being super contagious compared to the previously known strains has quickly spread across the globe. WHO declared the virus as a global pandemic on March 11, 2020. By then the epicenter of the virus had been shifted from China to Eastern Europe followed by the USA. As updated by the World Health Organization (WHO) on May 28, 2020, the COVID-19 outbreak has reached 213 countries and territories worldwide with 5,593,631 confirmed cases and 353,334 deaths (1).

The virus is yet to be peaked in most of the countries that got infected, and on top of that public health experts have warned that the newest epicenter of the virus could be shifted to the densely populated south and south-east Asia (2). Despite the concerted efforts by every stakeholder, tens of thousands of new cases of COVID-19 have been arising every day. Suddenly the world is facing a severe crisis of ICU beds and artificial respirators. From frontline doctors and nurses to pharmacists, all healthcare professionals are now facing an intense workload. It would take years to develop a vaccine for this novel coronavirus considering the fact that we are yet to see any effective vaccines for the previous coronavirus outbreaks (e.g., SARS & MERS) (3). Besides no well-established and specific anti-viral drug for this virus is available right now. Under this context, it is essential to better elucidate the clinical characteristics of the virus for a clear understanding of the disease and proper management of the patients by the health care providers. Moreover, the diagnosis also coincides with many challenges that include false test results, sampling errors, asymptomatic cases, insufficient testing facilities and lack of consciousness among mass people. Due to higher cost for RT-PCR testing facilities and lack of skilled manpower, many countries could not make it available across every regions, specially remote areas. In addition, due to the unwillingness of the patients arising out of fear of contamination and tendency to avoid procedural complexities, many suspected cases remain undiagnosed. To get the actual scenario of the prevalence and incidences as well as for the proper management of COVID-19 addressing all the diagnostic challenges is of utmost importance. This systematic review presents the summary of published reports up to 7th May 2020 on the clinical characteristics of COVID-19 for a better understanding of the frontiers team (especially to the medical doctors) involved in the treatment and management of COVID-19 patients. Additionally, the challenges for the prevention, treatment and management of COVID-19 have been discussed in the review.

## Methodology

### Protocol

This systematic review protocol is designed following the PRISMA (Preferred Reporting Items for Systematic Reviews and Meta-Analyses) Statement-2009 (4).

### Literature Search Strategy

We conducted a systematic and comprehensive search of important online databases—PubMed, Web of Science, Science Direct, Scopus, Wiley Online Library, and Google Scholar. The articles published and available online up to 7th May 2020 were considered to include in this study. The keywords used for the searches are included in the Table 1. All the searches were done in the English language only.

**Table 1:** Search strategy.

Database	PubMed, Web of Science, Science Direct, Scopus, Wiley Online Library, Google Scholar
Articles included	Articles published up to 7th May 2020 were included in the study
Keywords used for the search	Clinical characteristics, Clinical features, Clinical symptoms, Findings, Diagnosis, Novel coronavirus 2019, COVID-19, SARS-CoV-2, Challenges
Language used	English
Inclusion criteria	i) Articles containing clinical characteristics of COVID-19 ii) Original articles iii) Case series iv) Published in English language
Exclusion criteria	i) Reviews ii) Meta-analysis iii) Case reports iv) Expert opinions v) Newspaper articles vi) Commentaries vii) Prospective viii) Correspondence

### Eligibility Criteria

The search results were subjected to a range of inclusion and exclusion criteria, as listed in Table 1. The inclusion criteria mainly include original peer-reviewed articles (articles based on direct clinical data of the COVID-19 patients in various clinical settings). We opted out case reports, meta-analyses, expert opinions, newspaper articles, and commentaries.

### Screening and Study Selection

**Figure 1:**
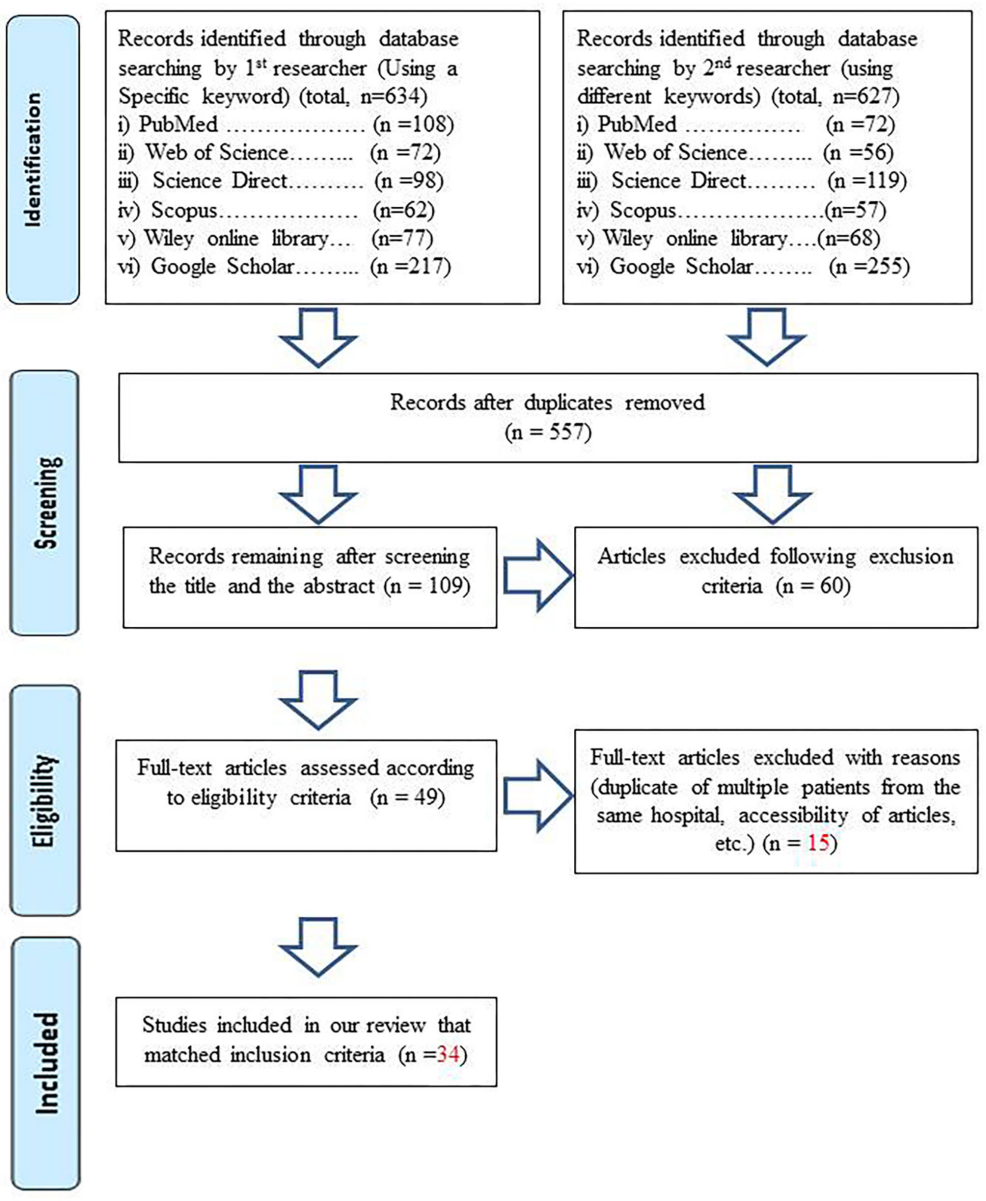
PRISMA (Preferred Reporting Items for Systematic Reviews and Meta-Analyses) for the identification and screening of articles to include in the study according to eligible criteria.

Following the eligibility criteria, two researchers were collaboratively involved in the screening and selection procedure of the articles of interest. The screening procedure was based on the PRISMA-2009 flow diagram, as presented in Figure 1. To present a global overview of the clinical trends of COVID-19, we have selected original studies from different hospitals across the world. The time period of each study and other related data were carefully screened to avoid any duplicates. Multiple studies from the same institutions were mainly analyzed and included avoiding data duplication. Observational studies reporting desired clinical parameters like symptoms, laboratory characteristics, and risk factors were included in the review. Case reports were discarded because they do not add significant value when stacked up against case studies. The whole procedure was overseen by two experienced researchers (an expert clinician and supervisor of this project).

### Data Extraction and Entry

The full text of the selected articles was thoroughly examined for relevant data of this systematic review that included the author's name, name of the hospital where the research had been conducted, number of case-patients and their clinical features, etc. We divided the case studies into three categories, namely, (i) Case studies in Hubei province only (China) (ii) Case studies in China (outside Hubei) (iii) Global case studies outside China (Table 2). We recorded and summarized the extracted data in Microsoft Excel. The whole process was carefully monitored and adjusted, had there been any discrepancies, by two independent expert researchers, including a clinician.

**Table 2:** Studies included in the review.

**Region**	**References**	**Journal**	**Hospital/Region**	**Total case patients *(n)***
Case studies in China (inside Hubei)	(5)	Allergy: European Journal of Allergy and Clinical Immunology	No.7 hospital of Wuhan	140
	(6)	SSRN Electronic Journal	Zhongnan Hospital of Wuhan University	221
	(7)	Clinical Infectious Diseases	Union hospital in Wuhan	69
	(8)	The Lancet	Outpatients of 30 hospitals in Wuhan designated for covid-19 treatment	124
	(9)	The Lancet Respiratory Medicine	Wuhan Jin Yin-tan hospital	52
	(10)	SSRN Electronic Journal	Renmin Hospital of Wuhan University	101
	(11)	Journal of the American Medical Association	Department of Critical Care Medicine, Zhongnan Hospital of Wuhan University	138
	(12)	The Lancet	Jin Yin-tan Hospital, Wuhan	41
	(13)	Chinese Medical Journal	9 tertiary hospitals of Hubei Province	137
	(14)	The Lancet	Jinyintan Hospital in Wuhan, China	99
	(14)	MedRxiv	Mobile Cabin Hospital of Optical Valley and Tongji Hospital of Huazhong University of Science and Technology in Wuhan	534
Case studies in China (outside Hubei)	(15)	Investigative Radiology	N/A	80
	(16)	Journal of Infection	57 hospitals across Beijing	262
	(17)	Journal of Infection	Wenzhou central hospital,Ruian people's hospital, Yueqing people's hospital	149
	(18)	China	Suzhou Fifth People's Hospital	69
	(19)	Clinical infectious diseases.	3 Grade IIIA hospitals of Jiangsu	80
	(20)	The BMJ	7 hospitals in Zhejiang	62
	(21)	Journal of Infection	Shanghai Public Health Clinical Center (SPHCC), Shanghai, China	249
	(22)	European Journal of Radiology	6 hospitals across Anhui province	73
	(23)	European journal of nuclear medicine and molecular imaging	Guangzhou Eighth People's Hospital	90
	(24)	European Respiratory Journal	First Affiliated Hospital of Zhengzhou University	18
	(14)	MedRxiv	5 hospitals across Zhejiang province	91
	(25)	European Review for Medical and Pharmacological Sciences	North Hospital of Changsha First Hospital	161
Global case studies outside China	(26)	MedRxiv	N/A	12
	(27)	Jama	Evergreen Hospital, USA	21
	(28)	Jama	12 hospitals in New York City, Long Island, and Westchester County, New York, within the Northwell Health system	5700
	(29)	Jama	72 hospitals of Lombardy ICU Network, Milan, Italy	1591
	(30)	The Lancet	N/A	17
	(31)	Journal of the American Medical Association	National Centre for Infectious Diseases, Singapore General Hospital, Changi General Hospital, and Sengkang General Hospital, Singapore	18
	(32)	International Journal of Biological Sciences	Centro Hospitalar Conde de São Januário, Macau	10
	(33)	Influenza and Other Respiratory Viruses.	Castle Hill Hospital, UK	68
	(34)	Travel Medicine and Infectious Disease	The Mediterranean Infection University Hospital Institute, France	280
	(35)	The Lancet	Self-Defense Forces Central Hospital, Japan	104
	(36)	Osong Public Health Res Perspect	N/A	28

### Quality Assessment

The validation instrument called “methodological index for non-randomized studies” (MINORS) was used to assess the methodological quality and bias risk of our data (Table 3). It is based on eight criteria for non-comparative clinical reviews, and the global acceptance score had been set to 16 (37).

**Table 3:** Quality ratings of included studies according to MINORS^*^.

	**References**	**①**	**②**	**③**	**④**	**⑤**	**⑥**	**⑦**	**⑧**	**Total score**	**Overall**
											**rating**
Case studies in China (inside Hubei)	(5)	2	2	2	2	2	1	2	1	14	Good
	(6)	2	2	2	2	2	0	2	2	14	Good
	(7)	2	2	2	2	2	1	2	0	13	Good
	(8)	2	2	2	2	2	1	2	1	14	Good
	(9)	2	2	2	2	2	2	2	0	14	Good
	(10)	2	2	2	2	2	0	1	1	12	Satisfactory
	(11)	2	2	2	2	2	0	2	1	13	Good
	(12)	2	2	2	2	2	2	1	0	13	Good
	(13)	2	2	2	2	2	1	2	1	14	Good
	(14)	2	2	2	2	2	2	2	0	14	Good
	(14)	2	2	2	2	2	0	2	2	14	Good
Case studies in China (outside Hubei)	(15)	2	2	2	2	2	2	1	0	13	Good
	(16)	2	2	2	2	2	1	2	2	15	Excellent
	(17)	2	2	2	2	2	1	1	1	13	Good
	(18)	2	2	2	2	2	1	1	0	12	Satisfactory
	(19)	2	2	2	2	2	2	2	0	14	Good
	(20)	2	2	2	2	2	2	2	0	14	Good
	(21)	2	2	2	2	2	0	2	2	14	Good
	(22)	2	2	2	2	2	1	2	0	13	Good
	(23)	2	2	2	2	2	2	2	0	14	Good
	(24)	2	2	2	2	2	1	2	0	13	Good
	(14)	2	2	2	2	2	1	2	1	14	Good
	(25)	2	2	2	2	2	1	1	1	13	Good
Global case studies outside China	(26)	2	2	2	2	2	0	1	0	11	Satisfactory
	(27)	2	2	2	2	2	1	2	0	13	Good
	(28)	2	2	2	2	2	1	1	0	12	Satisfactory
	(29)	2	2	2	2	2	1	1	0	12	Satisfactory
	(30)	2	2	2	2	2	0	1	0	11	Satisfactory
	(31)	2	2	2	2	2	2	2	0	14	Good
	(32)	2	2	2	2	2	1	1	0	12	Satisfactory
	(33)	2	2	2	2	2	1	2	0	13	Good
	(5)	2	2	2	2	2	2	1	2	15	Excellent
	(6)	2	2	2	2	2	0	1	1	12	Satisfactory
	(7)	2	2	2	2	2	1	2	0	13	Good

* *①A clearly stated aim; ②Inclusion of consecutive patients; ③Prospective collection of data; ④Endpoints appropriate to the aim of the study; ⑤Unbiased assessment of the study endpoint; ⑥Follow-up period appropriate to the aim of the study; ⑦Loss to follow up less than 5%; ⑧Prospective calculation of the study size*.

*The items are scored 0 (not reported), 1 (reported but inadequate) or 2 (reported and adequate). The global ideal score being 16 for non-comparative studies. The scoring was characterized as follows: 15-16, Excellent, 13-14, good, 11-12, Satisfactory, 9-10, Weak, <9, Unacceptable*.

## Results

### Study Selection and Characterization

The result of our screening and selection protocol has been shown in Figure 1. We initially retrieved 557 articles after removing duplicates that were later shortened to 34 following the screening protocol for the selection of eligible articles. The relevant information of these finally selected articles along, with associated hospital or medical institution names, has been given in Table 2. The total number of case-patients in these studies is 10,889. We retrieved a total of 176 variables regarding these patients that included demographic features, laboratory findings, symptoms, comorbidities, etc. (Tables 4–**9**) from these articles for the review. Most of the studies were from China, particularly from the Hubei province. For convenience, the data were split into three groups as mentioned earlier. But we inferred our final results after combining them into a single dataset.

**Table 4:** Demographic and epidemiologic features.

**Region**	**Global (Excluding China)**	**Only Hubei (China)**	**Outside Hubei (China)**	**Total/Mean**	**Percentage (%) on total**
Number of studies	11	11	12	34	–
Number of case patients	7849	1656	1384	10889	–
**Demographic features**					
Median Age (in years) (Range)	51.3 (0.5–84)	55.6 (16–87)	45.1 (1–94)	49.8 (0.5–94)	–
**Sex**					
Male	5000/7849	867/ 1656	700/1384	6567/10889	60.3
Female	2849/7849	789/1656	684/1384	4422/10889	39.7
Smoking (current)	577/3683	38/767	26/80	641/4530	14.2
Severity on diagnosis/admission
Severe/ ICU/ critical patient	2042/4640	212/592	154/1214	2408/6446	37.4
Non-severe/non-ICU patient	2598/4640	380/592	1060/1214	4038/6446	62.6
**Epidemiological Data (Transmission Pattern)**					
Travel/residence history in Wuhan	53/365	–	411/816	464/1181	39.2
Contact with people from Hubei Province/stay in Hubei	–	–	210/238	210/238	88.2
Contact with COVID-19 patient in family or community	10/292	395/1486	275/649	680/2427	28
No obvious contact history with COVID-19 patient	–	–	8/254	417/667	62.5
Resident of the infected area	280/347	–	137/320	19/308	6.2
Contact with person with fever	19/308	–	–	159/1486	10.70
Infection during hospitalization	–	159/1486	–	43/1486	2.89
Hospital staff	-	43/1486	-	43/1486	2.89
Huanan seafood wholesale market exposure	-	43/1486	-	118/1486	7.94
No obvious contact history with COVID-19 patient	-	118/1486	-	8/254	3.1

### Demographic Characteristics

The mean age of the case population was 50.6 years (0.5–94). The percentage of the male population was 60.3% (6567/10889), whereas that of the female population was 39.7% (4322/10889). The case population includes Asian, European and North American patients. Around 14.2% (641/4530) of them were found to be current smokers (Table 4).

### Clinical Symptoms

The most frequently observed clinical symptoms (Table 5) of the COVID-19 patients were cough/ dry cough 59.6 (2146/3598), fever 46.9 (4342/9242), fatigue 27.8 (1000/3598), dyspnea/shortness of breath 20.23 (728/3598), muscle ache/myalgia 12.64 (455/3598), diarrhea 11.95 (430/3598), headache 10.8 (389/3598), anorexia 9.9 (356/3598), sore throat 7.5 (270/3598), expectoration 7.48 (269/3598), upper airway congestion 6.67 (240/3598), and rhinitis 5.86 (211/3598). The lesser observed symptoms were pneumonia 2.89% (104/3598), abdominal Pain 2.31% (83/3598), nausea/vomiting 2.28 (82/3598), pharyngitis/pharyngalgia 1.83 (66/3598), chest pain 1.81 (65/3598), dizziness 1.56 (56/3598), chest tightness 1.25 (45/3598), malaise 0.61 (22/3598), chill 0.78 (28/3598), hemoptysis 0.42 (15/3598), heart palpitations 0.28 (10/3598), confusion 0.25 (9/3598), ARDS 0.22 (8/3598), belching 0.2 (7/3598), back discomfort 0.1 (3/3598), and arthralgia 0.03 (1/3598). A minor portion, 0.56% (20/3598), of the patients of the case population was found to be asymptomatic as well.

**Table 5:** Clinical symptoms of COVID-19 patients.

**Symptoms**	**Global (Excluding China) (symptomaic patients/case patients)**	**Only Hubei (China) (symptomatic patients/case patients)**	**Outside Hubei (China) (symptomatic patients/case patients)**	**Patients with symptoms/Total no. of case patients**	**Percentage (%) on total**
Cough/dry cough	338/558	1016/1656	792/1384	2146/3598	59.6
Fever	2020/6202	1200/1656	1122/1384	4342/9242	46.9
Highest temperature (°C)	37.34 (35.3–39.2)	38.5 (37.6–39.14)	39.0(36.99–41)	38.3 (35.3–41)	–
Fatigue	3/558	679/1656	318/1384	1000/3598	27.8
Dyspnea/shortness of breath	70/558	542/1656	116/1384	728/3598	20.23
Muscle ache/myalgia	75/558	287/1656	93/1384	455/3598	12.64
Diarrhea	29/558	284/1656	117/1384	430/3598	11.95
Headache	76/558	189/1656	124/1384	389/3598	10.8
Anorexia	–	305/1656	51/1384	356/3598	9.9
Sore throat	84/558	114/1656	72/1384	270/3598	7.5
Rhinitis/rhinorrhea	186/558	–	25/1384	211/3598	5.86
Upper airway congestion	22/558	218/1656	–	240/3598	6.67
Expectoration/sputum production	–	95/1656	174/1384	269/3598	7.48
Pneumonia	21/558	7/1656	76/1384	104/3598	2.89
Abdominal pain	2/558	74/1656	7/1384	83/3598	2.31
Nausea and vomiting	8/558	59/1656	15/1384	82/3598	2.28
Pharyngitis/pharyngalgia	–	57/1656	9/1384	66/3598	1.83
Chest pain	35/558	17/1656	13/1384	65/3598	1.81
Dizziness	2/558	18/1656	36/1384	56/3598	1.56
Chest tightness	–	–	45/1384	45/3598	1.25
Malaise	22/558	–	–	22/3598	0.61
Chill	1/558	–	27/1384	28/3598	0.78
Hemoptysis	–	9/1656	6/1384	15/3598	0.42
Heart palpitations	–	10/1656	–	10/3598	0.28
Confusion	–	9/1656	–	9/3598	0.25
ARDS	–	–	8/1384	8/3598	0.22
Belching	–	7/1656	–	7/3598	0.2
Back discomfort	–	–	3/1384	3/3598	0.1
Arthralgia	–	1/1656	–	1/3598	0.03
Others	3/558	–	–	3/3598	0.1
Asymptomatic	4/558	–	16/1384	20/3598	0.56

### Laboratory Findings

The laboratory findings (Table 6) included RT-PCR assay results, routine blood tests, and tests for various other blood-based biomarkers (e.g., coagulation factors, infection-related biomarkers, etc.). Among 8,650 patients, 8,253 (95.4%) were tested positive for SARS-CoV-2 in RT-PCR assay. Among the blood-based examinations, lymphocytopenia 55.9% (4177/7470) was most frequently observed. Other major lab findings include elevated levels of C-reactive protein 61.9% (830/1340), Aspartate aminotransferase 53.3% (3481/6537), Alanine aminotransferase 35.64% (2318/6503), lactate dehydrogenase 40.8% (392/973), ESR 72.99% (173/237), serum ferritin 63% (62/99), Interleukin-6 (IL-6) 52% (51/99), prothrombin time 35.47% (102/286), and D-dimer 28.06% (179/638). On the contrary, the levels of platelets 17.26% (160/927), eosinophils 59.0% (121/205), hemoglobin 29% (125/431), albumin 38.4% (187/487), and blood urea nitrogen (BUN) 20.9% (19/91) were reported to decrease.

**Table 6:** Laboratory findings and physical examinations.

**Laboratory findings**	**Global (Excluding China)**	**Only Hubei (China)**	**Outside Hubei (China)**	**Total/Mean (Range)**	**Total percentage (%)**
SARS-CoV-2 RT-PCR assay + (%)	7147/7330	928/1139	178 / 181	8253/8650	95.4
**Blood Routine**					
Leukocytes (× 10^9^/L, normal range 3.5–9.5)	8.3 (1.7–16.9)	6.1 (3.2–13)	4.7 (2.48–6.95)	6.4 (1.7-16.9)	-
Increased–No./total No. (%)	5/68	103/702	38/637	146/1407	10.38
Decreased–No./total No. (%)	21/114	206/702	119/637	346/1453	23.81
Neutrophils (× 10^9^/L, normal range 1.8–6.3)	2.65 (0.7–4.5)	4.6 (1.62–9.13)	3.3 (2.0–5.9)	3.52 (0.7-9.13)	-
Increased–No. /total No. (%)	-	84/290	64/485	148/775	19.1
Decreased–No. /total No. (%)	1/10	-	52/ 389	53/399	13.28
Lymphocytes (× 10^9^/L, normal range 1.1–3.2)	1.1 (0.2–1.7)	0.85 (0.6–1.46)	1.1 (0.4–1.66)	1.02 (0.2–1.7)	–
Increased–No./total No. (%)	-	-	70/222	70/222	31.5
Decreased–No./total No. (%)	3407/5745	551/1017	219/708	4177/7470	55.9
Platelets (× 10^9^/L, normal range 125–350)	158 (116–217)	176 (127–263)	173.9 (78.25–238)	169.7 (78.25–263)	-
Increased–No./total No. (%)	-	14/223	14/382	28/605	4.63
Decreased–No./total No. (%)	12/121	37/263	111/543	160/927	17.26
Monocytes (%, normal range 3–8)	2.83 (0.5–12.22)	0.37 (0.23–0.55)	0.44 (0.27–0.7)	1.21 (0.23–12.22	–
Increased–No./total No. (%)	-	-	41/251	41/251	16.3
Decreased–No. /total No. (%)	-	-	1/171	1/171	0.6
Hemoglobin (g/L, normal range 130–175)	12.67 (8–17.2)	130 (118–146)	131.8 (120–152.3)	92.5 (8–152.3)	-
Decreased–No./total No. (%)	–	50/99	75/332	125/431	29
Eosinophils (× 10^9^/L, normal range 0.02–0.52)	–	0.011 (0.00–0.05)	–	0.011 (0.00–0.05)	–
Decreased–No./total No. (%)	–	121/205	–	121/205	59.00
**Blood biochemistry**					
Aspartate aminotransferase (U/L, normal range 15–40)	33.33 (9.0–71)	31.2 (22–48)	25.3 (15.75–39)	29.9 (9.0–71)	–
Increased–No. /total No. (%)	3283/5700	69/209	129/628	3481/6537	53.3
Decreased–No./total No. (%)	–	–	19/153	19/153	12.4
Alanine aminotransferase (U/L, normal range 9–50)	36.6 (19.3–55.0)	28.6 (16–53)	22.7 (12.0–39.5)	29.3 (12.0–55.0)	–
Increased–No. /total No. (%)	2197/5769	51/168	70/566	2318/6503	35.64
Decreased–No./total No. (%)	–	–	8/240	8/240	3.3
Albumin (g/L, normal range 35–57)	–	32.04	39.8(5.48–46.3)	35.92(5.48–46.3)	–
Increased–No./total No. (%)	–	–	3/240	3/240	1.3
Decreased–No./total No. (%)	–	97/98	90/389	187/487	38.4
Creatinine (μmol/L, normal range 64–104)	83.3 (7.6–343.1)	72.7 (56–87)	69.4(51.28–90)	75.13(7.6–343.1)	–
Increased–No./total No. (%)	–	7/140	116/451	123/591	20.8
Decreased–No./total No. (%)	–	21/99	81/371	102/470	21.7
Serum Creatinine Kinase (mmol/L, normal range 40–200)	117.67 (45–1290)	90.1 (51–219)	81.5(40.5–191)	96.42(40.5–1290	–
Increased–No./total No. (%)	1/10	30/199	35/291	66/500	13.2
Decreased–No./total No. (%)	–	23/99	76/211	99/310	31.9
Lactate dehydrogenase (U/L, range12–250)	381.3 (206.5–796)	266.8 (174–447)	246.02(94.5–554)	298.04(94.5–796)	–
Increased–No. /total No. (%)	33/114	184/338	179/521	392/973	40.8
Glucose (mmol/L; normal range 3·9–6·1)	–	8.2	6.3(1.97–7.7)	7.3(1.97–7.7)	–
Increased–No./total No. (%)	–	51/99	78/229	129/328	39.3
Decreased–No./total No. (%)	–	–	1/149	1/149	0.7
Total Bilirubin (μmol/L, normal value 4.0–17.1)	8.13 (4–18.81)	14.8 (8.4–31.7)	9.3 (5.4–15.43)	10.74 (4–31.7)	–
Increased–No. /total No. (%)	–	18/99	25/459	43/558	7.71
Decreased–No./total No. (%)	–	–	9/218	9/218	4.1
Blood urea nitrogen (mmol/L; normal range 2.8–7.6)	–	6.6 (3.4–13.03)	4.4(3.3–5.9)	5.5(3.3–13.03	–
Increased–No. /total No. (%)	–	–	3/171	3/171	1.8
Decreased–No./total No. (%)	–	–	19/91	19/91	20.9
Lactate concentration (mmol/L normal range 0.5–1.6)	1.5 (1.1–2.1)	1.5 (0.7–3.2)	1.4(1.1–2.1)	1.47(0.7–3.2)	–
Hematocrit (%, normal range M: 40–50, F: 37–48)	12.67 (8–17.2)	40.3 (36.5–43.5)	–	40.3 (36.5–43.5)	–
**Coagulation function**	–	–	–	–	–
Activated partial thromboplastin time in seconds (normal range 21–37)	–	27.3	26(17.2–38.27)	26.65 (17.2–38.27)	–
Increased–No. /total No. (%)	–	6/99	40/149	46/248	18.55
Decreased–No./total No. (%)	–	16/99	2/80	18/179	10.05
Prothrombin time in seconds (9.4–12.5)	–	12.6 (10.1–17.39)	11.5(9.3–13.6)	12.05 (9.3–17.39)	–
Increased–No. (>1.00)/total No. (%)	–	85/137	17/149	102/286	35.47
Decreased–No./total No. (%)	–	30/99	7/229	37/328	11.28
D–dimer (ng/L; normal range 0–500)	438 (262–872)	712.2 (100–2800)	368 (106–2400)	506.1 (100–2800)	–
Increased–No./total No. (%)		113/249	56/389	179/638	28.06
**Infection–related biomarkers**					
Procalcitonin (PCT) (ng/ml, normal range 0–0.1)	0.7 (0.03–9.59)	0.24	0.35(0–2.6)	0.43(0.0–9.59)	–
Increased–No. (>1.00)/total No. (%)	–	125/677	58/329	183/1006	18.2
ESR (mm/h normal range 0–20)	–	20 (8–31)	32.95(9–90)	26.5(8–90	–
Increased–No. (≥20)/total No. (%)	–	114/157	59/80	173/237	72.99
Serum ferritin (ng/mL, normal range 21.0–274.7)	–	808.7	–	808.7	–
Increased–No. /total No. (%)	–	62/99	–	62/99	63.00
C–reactive protein (CRP) (mg/L, normal range <10)	30.94 (0.1–97.5)	37.8 (6.78–67.4)	10.3 (1.8–50.61)	26.35(0.1–97.5)	–
Increased–No./total No. (%)	17/96	379/508	434/736	830/1340	61.9
Interleukin−6 (pg/mL, normal range 0.0–7.0)	–	–	0/181	7.9 (6.1–10.6)	–
Increased–No. /total No. (%)	–	7.9 (6.1–10.6)	–	51/99	52.00
Decreased–No./total No. (%)	–	51/99	–	0/181	0
**Physical Examinations**					
Respiratory rate, breaths/min. (Normal range: 12–20)	16.33 (13.5–21)	20.5 (19–30)	22.5	19.8(13.5–30.0)	–
O_2_ saturation,% (Normal range: 75–100)	96.9 (91–100)	–	91.78	94.34(91–100)	–
Mean Systolic pressure (mmHg) (Normal range−100)	128 (96–180)	91.5 (83–105)	114.5 (80–145.42)	111.3(80–180)	–
Heart rate /min (Normal range: 60–100)	92.4	–	88.44	90.42(52–125)	–

### Radiological Findings

The results of chest CT scans (Table 7) showed abnormality of at least one kind in 93.5% (1668/1785) of the case-patients. The predominant abnormality had been bilateral lungs found in 71.1% (1581/2223) of the patients. Other significant findings of the lung characteristics from CT scan result include Ground-glass opacity 48% (432/900), consolidation 21.88% (140/640), pleural effusion 20.6% (195/947), the lesion in lung 78.3% (180/230), enlargement of lymph node 50.7% (153/302), thickening of bronchial wall 30.3% (80/264), thickening of lung texture 84.9% (62/73), and thickening of Interlobular septal 47.1% (80/170).

**Table 7:** Radiological findings (Chest CT scan results).

**Radiological findings**	**Global (Excluding China) (n)**	**Only Hubei (China) *(n)***	**Outside Hubei (China) *(n)***	**Total *(n)***	**Percentage (%) in total**
**Chest CT**					
Normal	49/164	22/1292	38/329	109/1785	6.11
Abnormality	115/164	1262/1292	291/329	1668/1785	93.5
Unilateral lungs		144/1292	137/306	281/1598	17.6
Bilateral lungs	57/122	1187/1529	337/572	1581/2223	71.1
**Density and Inner Features**					
Ground–glass opacity	53/112	69/236	310/552	432/900	48
Consolidation	32/112	25/137	83/391	140/640	21.88
Mixed	–	–	35.4/132	35.4/132	26.8
**Other Features**					
Pleural effusion	6/21	31/534	158/392	195/947	20.6
Lung lesion	–	–	180/230	180/230	78.3
Interlobular septal thickening	–	–	80/170	80/170	47.1
Crazy paving pattern	–	–	34/170	34/170	20
Spider web sign	–	–	20/80	20/80	25
Sub–pleural line	–	–	16/80	16/80	20
Bronchial wall thickening	5/21	–	75/243	80/264	30.3
Lymph node enlargement	–	–	153/302	153/302	50.7
Pericardial effusion	–	–	5/170	5/170	2.9
Paving stone sign	–	–	25/73	25/73	34.25
Thickening of lung texture	–	–	62/73	62/73	84.9
Pulmonary edema	2/21	–	–	2/21	9.5
Venous congestion	1/21	–	–	1/21	4.8
Atelectasis	1/22	–	–	1/22	4.55

### Physical Examinations

The results of the physical examinations (Table 6) that included respiratory rate 19.8 breaths/min (13.5–30.0), pulse oximeter O_2_ saturation 94.34%, mean systolic pressure 111.3 mmHg (80–180), and heart rate 90.42/min (52–125) were found to be slightly higher than their respective normal range.

### Comorbidities

Various underlying medical conditions (Table 8) were found in the case-patients, the most significant ones being hypertension 35.9% (3909/10889), diabetes 20.17% (2196/10889), obesity 15.95% (1735/10889), cardiovascular disease 13.92% (1516/10889), asthma 4.42% (481/10889), COPD 4.31% (469/10889) and malignancy 3.99% (435/10889). Several patients were also found to be co-infected with other viruses, 9.1% (244/2684), Bacteria 4.99% (24/481), and Fungus 3.43% (11/320).

**Table 8:** Comorbidities.

**Comorbidities**	**Global (Excluding China) (*n* = 7849)**	**Only Hubei (China) (*n* = 1656)**	**Outside Hubei (China) (*n* = 384)**	**Total (*n* = 10889)**	**Percentage (%) in total**
Hypertension	3541/7849	296/1656	72/1384	3909/10889	35.9
Diabetes	2003/7849	160/1656	33/1384	2196/10889	20.17
Obesity (BMI ≥30)	1737/7849	–	–	1737/10889	15.95
Cardiovascular disease	1231/7849	164/1656	121/1384	1516/10889	13.92
Asthma	481/7849	–	–	481/10889	4.42
COPD	344/7849	110/1656	15/1384	469/10889	4.31
Malignancy/cancer	401/7849	25/1656	9/1384	435/10889	3.99
Chronic renal insufficiency	304/7849	26/1656	4/1384	334/10889	3.07
End-stage kidney disease	188/7849	–	–	188/10889	1.73
Hyperlipidemia	192/7849	–	–	192/10889	1.76
Cerebrovascular Disease	–	46/1656	116/1384	162/10889	1.49
Pneumonia	–	7/1656	76/1384	83/10889	0.76
Chronic liver disease	40/7849	31/1656	15/1384	86/10889	0.79
Chronic gastritis and gastric ulcer	0/7849	47/1656	11/1384	58/10889	0.53
History of solid organ transplant	57/7849	–	–	57/10889	0.52
Fatty liver and abnormal liver function	5/7849	43/1656	–	48/10889	0.44
Endocrine diseases	–	5/1656	39/1384	44/10889	0.40
HIV infection	43/7849	2/1656	–	45/10889	0.41
ARDS	8/7849	17/1656	2/1384	27/10889	0.25
Acute kidney injury	2/7849	22/1656	–	24/10889	0.22
Autoimmune disease	1/7849	10/1656	–	11/10889	0.1
Immunosuppression	3/7849	3/1656	3/1384	9/10889	0.08
**Co-infection**					
Other viruses	211/2364	33/320	–	244/2684	9.10
Bacteria	1/21	23/460	–	24/481	4.99
Fungus	–	11/320	–	11/320	3.43

### Clinical Progression Data

The median incubation period of the patients is found to be 5.36 days (1.5–15) based on 12 studies involving 1080 patients. The median hospital stay of the patients is eight based on three studies. Median days from onset of illness to hospital admission is 4.83 (0–11) based on 13 studies. Global (outside China) case fatality rate was found to be 22.24% (969/4357), among which 21.24% (564/2655) was in the USA, 25.61% (405/1581) in Italy, and 0% (0/121) was in other countries (Singapore and Diamond Princess Cruise Ship while it was staying in Japan). In comparison the case fatality inside the Hubei Province of China was 11.71% (194/1656) (Table 9).

**Table 9:** Disease progression and other clinical data.

**Disease progression and other clinical data**	**Global (Excluding China)**	**Only Hubei (China)**	**Outside Hubei (China)**	**Total/Mean (Range)**	**Percentage (%)**
Median hospital stay of patients (days)	–	8	–	8	–
Median Incubation Period (days)	4.05 (2–9)	6 (5–13)	6.3 (1.5–15)	5.36 (1.5–15)	–
Days from onset of illness to dyspnea	4 (1–20)	–	–	4 (1–20)	–
Days from onset of illness to ICU	4.7 (1–14)	–	–	4.7 (1–14)	–
Days from Onset of symptoms to hospital admission	4 (0–11)	7 (4–11)	3.5 (0.8–8.2)	4.83 (0–11)	–
Days From hospital admission to death	–	4 (2–7)	–	4 (2–7)	–
Days from onset of illness to ARDS	–	8 (6–12)	–	8 (6–12)	–
Length of follow-up days	7.6 (1–15)	–	–	7.6 (1–15)	–
Total Death (Case Fatality Rate)^*^	Global: 969/4357 (22.24%) [USA: 564/2655 (21.24%), Italy: 405/1581 (25.61%), Other countries (Singapore, and Diamond Princess Cruise Ship while it was staying in Japan: 0/121(0%)]	194/1656 (11.71%)	5/893 (0.56%)	1168/6906	16.9

*ARDS, Acute respiratory distress syndrome; ICU, intensive care unit*.

* *Based on the available follow-up data on patients from the selected case studies*.

### Exposure Pattern

A significant portion of the study cases has their exposure history linked to family members or their respective community 28% (680/2427) (Table 4) which is particularly the true for the study patients from China. Our review found that the exposure pattern in the global arena (outside China) has been largely centered on the imported cases 80.7% (280/347). This review also revealed that the hospital staff's exposure risk is 2.89% (43/1486) and the in-patients of the hospitals 2.89% (43/1486).

### Age-Dependent Effects of COVID-19

It is assumed that, the age of the patients may influence the clinical features, disease progression, complexities, and fatality of the COVID-19. However, we have analyzed and included the available data on this aspect. The notable findings include faster disease progression, higher mortality rate, progressively lower lymphocyte count, higher ICU admission rate, higher mortality rate for those receiving mechanical ventilation, and higher risk of severe heart attack among the older patients (Table 10).

**Table 10:** Age dependent effects of COVID-19.

**Parameter**	**Age related impact**	**References**
Severity of disease	Median age of severe patients (62.0) were significantly older than non-severe ones (51.0)	Zhang et al. (6)
Disease progression	Disease progressed rapidly in older patients with ARDS and septic shock	Zhang et al. (6)
Survivors vs. non-survivors	Non-survivors were older (mean age: 64.5 years) compared to the survivors (mean age: 51.9 years) Mortality rate increases in older (<65 years) patients with comorbidities	Yang et al. (17)
Severe heart attack/death	Older patients (>70 years) with chronic medical conditions are likely to suffer a severe heart attack and death.	Shi et al. (10)
ICU admission	Patients treated in ICU were significantly older (median age: 66 years) than patients not treated in ICU (median age: 51 years)	Wang et al. (11)
Mortality rate of the patients who received mechanical ventilation	Mortality rate were found to be higher (97.2%) in older patients (>65 years) compared to 76.4% in case of younger patients (18–65 years).	Richardson et al. (28)
Lowest absolute lymphocyte count	Progressively lower for older patients	Grasselli et al. (29)
Median fraction of inspired oxygen (FiO2)	Lower (60%) in younger patient compared to the older patients (70%)	Grasselli et al. (29)

## Discussion

This systematic review is based on a random effect model. As such, it contains data from studies that took place in different countries, including China, the USA, Japan, South Korea, France, and Singapore. All the studies were published in peer-reviewed articles with patient data between late December 2019 and May 7, 2020.

We managed to retrieve data on clinical characteristics of COVID-19 from 10,889 infected patients. Various studies and reports worldwide showed that COVID-19 seems to infect the male population more frequently than it does to female population. Our review also corroborates this claim as 60.3% (6487/10889) of our case population are male and the rest 39.7% (4241/10889) are female. Previously older age had been predicted to be an essential factor for higher mortality in SARS and MERS patients (38). Several studies in our review have reported the same. During the initial transmission period, COVID-19 seemed to affect the elderly more, as reflected by the mean age of 49.8 years (0.5–94) of the case population included in the review attributing to the higher frequency of comorbidities observed among the elderly (39). But in the current situation, it seems that the virus can equally affect everyone irrespective of age. However, it is evident that the clinical characteristics and prognosis of the disease greatly vary among patients with different age groups. Patients over 60 years tend to show more severe clinical manifestations and relatively longer disease duration, meaning they would require more careful monitoring and more comprehensive medical interventions (40).

The Chinese Center for Disease Control and Prevention (CDC) reported that the vast majority (81%) of COVID-19 patients will develop only mild symptoms, and the rest develop severe illness i.e., they will require oxygen therapy (14%) or require ICU treatment (5%) (41). Usually, COVID-19 patients are diagnosed with signs of severe pneumonia. Our review found that the most commonly reported symptoms are dry cough (59.6%), fever (46.9%), fatigue (27.8%), and dyspnea/shortness of breath (20.23%). These symptoms, together with prior contact history with suspected patients, immediately would require medical attention. Other less frequent symptoms include myalgia, diarrhea, headache, anorexia, sore throat, rhinitis, upper airway congestion, expectoration, pneumonia, etc. The symptoms are similar to those of SARS and MERS (42, 43). In most cases, the symptoms are mild during the initial days of infection but can be very severe, particularly for the elderly and patients with underlying respiratory diseases. Unlike SARS and MERS, SARS-CoV-2 behaves mildly during the initial stage of infection, making it significantly more contagious than the previous coronaviruses since the condition can go unnoticed in some cases (44). New shreds of evidence emerging from across the globe suggest that the asymptomatic cases of COVID-19 infections are rising (45, 46). But our review found that only a small percentage (0.56%) (20/3598) of the patients were asymptomatic which may be attributed to the fact that the initial tests for COVID-19 were only being conducted for patients with a distinct illness. To screen out the asymptomatic cases, experts have emphasized on comprehensive epidemiological investigations of the suspects by disease control specialists. Besides, the asymptomatic patients have shown consistent abnormalities in their CT scan reports for which radiological investigations can be a useful diagnostic tool to find the asymptomatic variants (47).

The most notable laboratory finding of our review is lymphocytopenia found in 55.9% cases (4177/7470). It was also reasonably expected in the influenza virus (H5N1), SARS, and MERS (48). In H5N1 influenza the fall in lymphocyte count had been attributed to dendritic cell (DC) dysfunction suggesting that a similar mechanism related to dysfunctional adaptive immunity can cause the same in COVID-19 patients (49). Besides, another study has demonstrated the correlation between the lymphocytopenia and the clinical severity of SARS-CoV-2 infection. The study also infers that the lymphocytopenia might be an outcome of the death of lymphocytes or the damage of lymphatic organs like thymus or spleen directly by the virus itself (25).

Another significant finding of our review is the elevated level of CRP 61.9% (830/1340) which supports a study that demonstrated elevated CRP levels in COVID-19 patients even though they did not have any kind of coinfections (50). It is to be noted that the CRP level is usually increased in bacterial or viral infections. However, it does not demonstrate a significant elevation in the case of mild viral infections. Another study claimed the potentiality of CRP as a comprehensive predictive factor of COVID-19 prior to the changes in other inflammatory-related blood parameters e.g., leucocytes, lymphocytes, and neutrophils (5). Besides, CDC guidelines have also reported an elevated CRP level in COVID-19 patients with higher CRP levels indicative of the severity of the infection and poor prognosis (51). Previously it has been associated with Influenza H1N1 & H7N9 and the SARS epidemic (52, 53). In COVID-19 patients, this significantly elevated level of CRP can be explained by the excessive production of inflammatory cytokines due to immune response as well as the damage of the lung alveoli (50).

LDH level has been found to be increased by 40.8% (392/973) in COVID-19 patients. Although it is abundant in tissues, LDH level is low in blood circulation. Elevated LDH, as reported in SARS, reflects tissue necrosis corresponding to hyperactive immunity (54). Besides, viral infections can cause lung tissue damage, which in turn raises the LDH level in blood circulation (55).

Our review revealed abnormal liver function tests in the case of patients with elevated ALT 36.65% (2318/6503) & AST 53.3% (3481/6537) level and lower albumin (38.4%) (187/487) and serum creatinine kinase level (31.9%) (99/310). One report has proposed that the hepatic dysfunction of COVID-19 patients can be linked to direct liver injury via viral hepatitis or due to abnormal levels of blood coagulative and infection-related functions, such as, elevated prothrombin time, D-dimer level, and serum ferritin, as found in our review (56).

We could not find sufficient data on the immunological parameters of the case patients. But one of the studies included in our review reported a higher IL-6 count in 51 (50%) of 99 patients (14). Besides, two recent studies have shown that IL-6 level was substantially increased in both severe and moderate patients. In addition to that, other pro-inflammatory parameters like IL10, IL2, IFN-γ, and TNF-α were found to be higher in more severe patients than in the moderate ones (21, 22). The magnitude of the cytokine storm or, more particularly, elevated IL-6 level has been associated with disease severity (13). It has been proposed as an essential parameter to predict respiratory failure risk based on a study that found a significantly higher IL-6 level in patients requiring ventilation (57).

The radiological findings showed that COVID-19 patients are accompanied by an abnormality in their CT scan report in 93.5% (1668/1785) cases. So CT scanning can provide an important base for early diagnosis of the virus. The typical CT scan features include bilateral lung 71.1% (1581/2223) and unilateral lung 17.6% (281/1598). The density characteristics of the lung lesions, found in 78.3% (180/230) cases, was mostly uneven, with ground-glass opacity 48% (432/900) as the primary presentation accompanied by consolidation in 21.88% (140/640) cases. CT scan reports have been demonstrated an association between disease progression and a higher rate of consolidative opacities (58). CT scan reports and the RT-PCR tests are generally found to be harmonious with a few exceptions. CT scan results can be a more specific diagnostic tool for the suspected COVID-19 cases since even the asymptomatic patients have shown abnormalities in their CT scan reports (46, 47). The less common CT findings include pleural effusion 20.6% (195/947), pericardial effusion 2.9% (5/170), bronchial wall thickening 30.3% (80/264), and interlobular septal thickening 47.1% (80/170) which have been reported as the disease progresses.

The median incubation period of the SARS-CoV-2 virus has been found to be 5.36 days (1.5–15). National Health Commission of China previously reported an incubation period of 1 to 14 days. The CDC has updated the mean incubation period to be between 2 to 14 days (51). However, various studies together with our findings suggest that the incubation period of SARS-CoV-2 is highly variable and requires more concrete statistically significant results. This high variability in the incubation period of SARS-CoV-2 has warranted a suitable quarantine period of at least 3 weeks to reduce the community transmission of the virus (59).

A significant portion of our case patients had one or more comorbidities indicating that a significant portion of our case patients is elderly. They are more likely to develop various chronic or acute clinical conditions on aging. Hypertension (35.9%) (3909/10889), diabetes (20.17%) (2196/10889), obesity (15.95%s) (1737/10889), cardiovascular diseases (13.92%) (1516/10889) and respiratory diseases (e.g., asthma-4.42%; COPD-4.31%) are the most frequent comorbidities found in the case patients of our review. The prevalence of comorbidities can be a major risk factor for severe patients compared to non-severe patients due to higher case fatality and poor prognosis (17). Thus, a proper medical history of the COVID-19 patients, particularly elderly patients, should be documented. Moreover, adequate clinical facilities should be made available for this group of patients.

Age-specific patient data was not sufficiently available in the case of most of our included studies. Still, for some crucial parameters, such as mortality rate and severity of the disease, a pattern was observed across the studies involving the older patients who, in general, possess a higher risk. Table 10 has listed all of the available age-related effects that we were able to retrieve. One of the recent studies has indicated that the children's overall risk factors are not significantly affected by the age and sex (60). Unlike adults, the children are at a lower risk of developing severe symptoms or death.

### Diagnostic Challenges of COVID-19

**Article search strategy:** For the collection of information on diagnostic challenges of COVID-19, we have extensively searched by using the keywords “COVID-19 and diagnostic challenges,” “challenges for the diagnosis of novel coronavirus diseases,” on Pubmed, Web of Science, Science Direct, Scopus, Wiley Online Library, Google Scholar databases and other online websites of reliable sources such as Gavi-The Vaccine Alliance (gavi.org), World Health Organization, US-FDA, etc.

### Review Findings on the Diagnosis Challenges of COVID-19

Every day the novel coronavirus is presenting us with new clinical challenges. As countries worldwide are considering to curb the limits on social distancing measures, general testing and rapid diagnosis of the disease have become of utmost importance. Even though various efforts are undertaken both by the government agencies and international bodies, the diagnosis of COVID-19 is still challenging. For convenience, the information on the current challenges for the diagnosis of COVID-19 have been summarized as follows:

1. Although RT-PCR has been regarded as the gold standard for viral detection of SARS-CoV-2, it also comes with few challenges. The equipment and lab facilities required for this testing can be expensive. The testing environment requires a certain biosafety level e.g., BSL-2 cabinets for sample preparation or ideally a negative pressure room (61). Most of the traditional testing centers all around the globe do not have these facilities. On top of that, these facilities require technicians with enough expertise and experience to perform the tests smoothly and flawlessly (62). Therefore, lack of testing centers and expert technicians are the primary diagnostic challenges faced by most of the countries all over the world. For this very reason, many countries failed to start coronavirus testing early in its transmission phase. Even when they had started testing, it was strictly restricted to suspected individuals with clear symptoms of the viral infection (63). Moreover, as countries all over the world are trying to increase their number of testing each day, this has put a serious pressure on the ready availability of reagents for the PCR reactions. Thus, shortage of testing kits is next big challenge to the rapid diagnosis of the disease.
2. Another crucial diagnostic challenge with RT-PCR testing is the risk of eliciting false-negative and false-positive results. It has been found that many patients with typical COVID-19 symptoms and CT features have shown a negative influence in RT-PCR testing (64). Therefore, a negative result should not preclude the patient as a COVID-19 suspect, and it should not be used as the only criterion of diagnosis. Besides, a false result due to human error cannot be overruled as under-trained technicians are performing many of these tests.
3. The collection of the patient sample (throat swab) presents another diagnostic challenge as this requires specific procedures to be followed for the sake of proper sample preparation for RT-PCR testing. Wrong sampling procedures can cause errors in RT-PCR testing (65). Moreover, a global shortage of personal protective equipment (PPE) has put additional challenges as adequate protective measures have to be made available for the staff involved in this sample collection procedure.
4. RT-PCR testing also faces another challenge that arises from the susceptibility of the viral mutations in the SARS-CoV-2 genome. Various studies have shown a rapid evolution of the virus (66, 67). Consequently, a false negative result may arise due to mutation in the primer or probe target regions (61). Although they are based on the most conserved regions of the viral genome, a slight variability can decrease the assay performance.
5. Asymptomatic patients have become a major challenge for both the clinicians and the administration. They are difficult to diagnose and isolate and they also pose a more significant threat of rapid and unchecked viral transmission. A recent study has found a similar viral load in asymptomatic patients compared to the symptomatic ones, indicative of their transmission potential (68). Another big concern is that most of these asymptomatic patients will not report to the hospitals or testing centers. Under such circumstances, their diagnosis solely depends on contact tracing or cluster screening by the disease control experts (68). Diagnosis of asymptomatic children is also challenging as their diagnosis is only limited to tracing their family history (69, 70). But this task is getting increasingly tricky both due to an overwhelming number of new cases arising each day and the limited workforce of the disease control centers.
6. Although COVID-19 patients have clear chest CT scan manifestations, several studies have reported usual CT scan reports despite being RT-PCR confirmed COVID-19 patients (9, 71). One study has found a false negative result of over 12% when attempted to predict the infection based on CT scan reports only (72). These reports clearly mean that clinicians have to be more vigilant in diagnosing the suspected cases of COVID-19 patients and consider multidimensional factors including laboratory parameters, CT scan reports, and other tests.
7. Additionally, most countries do not have a sufficient number of CT scan machines to support widespread testing of the rising number of COVID-19 patients (73). Besides, it takes a lot of effort to disinfect the machines after each test to prevent unwanted viral transmission.
8. Another challenge is that it requires multiple scans (at least 2, 6 days apart) for maximum accuracy in the diagnostic results meaning this would put additional pressure on the already scarce CT scan machines (74).
9. Sometimes suspected patients of the virus are showing unwillingness to come to test centers to confirm the presence of the virus. Some of the developing nations have reported these incidents, which were mostly attributed to the lack of concern, illiteracy, and the fear of social stigma (75). These patients remain undiagnosed for a considerable time till their symptoms get severe to the extent that they need hospitalization. So this group of patients presents us with a severe diagnostic challenge.
10. Serological detection tools utilize the detection of specific antibodies (IgM and IgG) against COVID-19 infections. These antibody tests have the advantage of low cost, fast detection and easy availability but suffer from low sensitivity as is seen with the antibody tests deployed for other coronavirus and influenza virus (76–78). However, IgM responses vary from person to person and require days to develop once they get infected. Considering that this may not be an useful tool for the accurate diagnosis of the viral infection except in confirming the late cases of COVID-19 and immunity of the recovered individuals (79). Due to these concerns, on April 1, 2020, the FDA granted Emergency Use Authorization (EUA). Still, they repeatedly expressed their doubts about the effectiveness of these tests in detecting the virus early during infection (80).

## Strength and Limitations

To our knowledge, this is one of the first attempts to summarize the clinical characteristics and diagnostic challenges of COVID-19 taking into considerations of all the available data from global studies and variabilities so that the researchers, health care workers, policy makers and related stakeholders can get a contemporary overview of the COVID-19 situation.

The most noticeable limitation of our review is the lack of consistent data on every variable across the studies that we have included, which happened due to underreporting of symptoms, comorbidities, laboratory results, or exposure patterns of the case patients. These initially published studies have some issues related to lack of adequate laboratory testing, which could be attributed to the fact that these studies had to be completed as fast as possible to keep people around the world up to date about the virus and to prepare them fast to manage the disease. Besides, we were able to fetch only a limited amount of data regarding the clinical progression and other epidemiologic characteristics that would otherwise come in handy in defining clinical characteristics. In addition, most of our studies were from China, as no data were available from other countries. It would be better to broaden the geographical scope of our review to get a more global scenario of the clinical characteristics of the outbreak.

## Conclusion

This systematic review summarized the latest clinical findings of the novel coronavirus outbreak that has virtually affected the whole world. To the best of our knowledge, this is the first large scale review comprising of studies from across the globe focusing on the clinical features of the COVID-19 patients. Laboratory tests and CT scan reports, together with clinical symptoms will provide useful information for the correct diagnosis and better management of the patients. This review provides a comprehensive overview and clear features of the clinical characteristics of COVID-19 which will help the physicians to make proper clinical decisions and correct assessment regarding the patients. Additionally, we have reviewed the challenges being faced for the diagnosis of the disease across the world. Overcoming these obstacles to the fast and prompt diagnosis of COVID-19 will be crucial for the proper containment of the disease.

## Data Availability Statement

All data generated or analyzed in this study have been included in the article. The raw data will be provided by the corresponding and/or by the first author on reasonable request.

## Author Contributions

SMHI and MMRS: conceived and designed the study; SMHI and PTR: Collected data through article search in different online databases; KAK and MMRS: Checked and verified collected data; SMHI, PTR, MMRS, FK, and SA: Analysis and interpretation of data; SMHI and MMRS: prepared the manuscript draft; AAT, CLP, INM, and LCM critically reviewed the manuscript. LCM was also involved with the planning of the study. All authors read the manuscript, agreed to be accountable for all aspects of the work, and approved the final manuscript.

## Conflict of Interest

The authors declare that the research was conducted in the absence of any commercial or financial relationships that could be construed as a potential conflict of interest.

## Abbreviations

COVID-19: Coronavirus diseases
Novel coronavirus 2019: 2019n-CoV
SARS-CoV-2: Severe Acute Respiratory Syndrome coronavirus 2
WHO: World Health Organization
MERS-CoV: Middle East Respiratory Syndrome Coronavirus
PRISMA: Preferred Reporting Items for Systematic Reviews and Meta-Analyses
CRP: C-Reactive Protein
ICU: Intensive Care Unit
MINORS: Methodical Index for Non-randomized Studies
CDC: Center for Disease Control and Prevention
LDH: Lactate dehydrogenase
INF: Interferon
TNF: Tumor necrosis factor
ESR: Erythrocyte sedimentation rate
ARDS: Acute respiratory distress syndrome
IL: *Interleukins*
*RT-PCR*: Reverse transcription polymerase chain reaction
BUN: Blood urea nitrogen
CT Scan: computed tomography scan.
